# Association of milk consumption with all-cause mortality and cardiovascular outcomes: a UK Biobank based large population cohort study

**DOI:** 10.1186/s12967-023-03980-4

**Published:** 2023-02-18

**Authors:** Jian Zhou, Ziyi Wu, Zhengjun Lin, Wanchun Wang, Rongjun Wan, Tang Liu

**Affiliations:** 1 Department of Orthopedics, The Second Xiangya Hospital of Central South University, Renmin Middle Road No. 139, Changsha, 410011 Hunan China; 2 Department of Respiratory Medicine, National Key Clinical Specialty, Branch of National Clinical Research Center for Respiratory Disease, Xiangya Hospital, Central South University, Changsha, 410008 Hunan China; 3 Laboratory of Bone Disorder, The Second Xiangya Hospital of Central South University, Changsha, 410011 Hunan China

**Keywords:** Milk type, Mortality, Cardiovascular outcome, Prospective study

## Abstract

**Background:**

The association of milk consumption with mortality and cardiovascular disease (CVD) outcomes was unclear.

**Objective:**

The present study was performed to reveal the association of full cream, semi-skimmed, skimmed, soy, and other milk with all-cause mortality and CVD outcomes.

**Methods:**

A prospective cohort study was performed using data from the UK Biobank. This study recruited 450,507 participants without CVD at baseline between 2006 and 2010 from UK Biobank and followed them up through 2021. Cox proportional hazard models were adopted to calculate the hazard ratios (HRs) and 95% confidence interval (CI) to understand the correlation between milk consumption and clinical outcomes. Subgroup and sensitivity analyses were further conducted.

**Results:**

Among the participants, 435,486 (96.7%) were milk consumers. Multivariable model indicated that the adjusted HR of association between milk consumption and all-cause mortality was 0.84 (95% CI 0.79 to 0.91; P = 0.000) for semi-skimmed milk; 0.82 (0.76 to 0.88; P = 0.000) for skimmed milk and 0.83 (0.75 to 0.93; P = 0.001) for soy milk. Semi-skimmed, skimmed, and soy milk use were significantly related to lower risks of CVD mortality, CVD event, and stroke.

**Conclusion:**

Compared with non-milk users, semi-skimmed milk, skimmed milk, and soy milk consumption were related to a lower risk of all-cause mortality and CVD outcomes. Among them, skim milk consumption was more beneficial for all-cause mortality, while soy milk consumption was more beneficial for CVD outcomes.

**Supplementary Information:**

The online version contains supplementary material available at 10.1186/s12967-023-03980-4.

## Introduction

Milk contains many essential nutrients, including calcium, phosphorus, and vitamin D. Many dietary guidelines commonly recommend milk in the diet [1–3]. Previous studies showed that the association of dairy milk consumption with mortality and cardiovascular disease (CVD) outcomes was highly controversial [1, 4–8]. Several reports showed a neutral association of dairy use with all-cause mortality and worse CVD outcomes [1, 4, 5, 9]. Meanwhile, a population-based cohort study demonstrated that dairy-related supplementation lowers the risk of CVD mortality [7]. Three prospective cohorts of U.S. healthcare professionals indicated that increased full cream milk use was associated with an increased risk of all-cause mortality [6]. However, several studies indicated that milk consumption was related to decreased all-cause mortality and CVD risk [8, 10]. Thus, the relationship of milk consumption with mortality and CVD outcomes is still unclear, and the different milk types' relationship with mortality is also less clear. A previous study reported that compared with those consuming whole milk, participants with reduced fat indicated a lower risk of mortality [11]. However, a cohort study showed that high milk consumption was connected to higher mortality [12]. Another study showed that intake of whole milk was related to a lower risk of CVD [13]. There is no direct evidence on whether semi-skimmed or skimmed milk was more beneficial to health than full-cream milk [14].

Large population-based cohort studies can provide additional information on the correlation of milk consumption with clinical outcomes, given the uncertainty. This prospective cohort study based on UK biobank cohort was conducted to explore the connection between intake of different types of milk, which including full cream, semi-skimmed, skimmed, soy and other milk type, and all-cause mortality and CVD outcomes and investigate the correction factors affecting these associations.

## Methods

### Study population

**Fig. 1:**
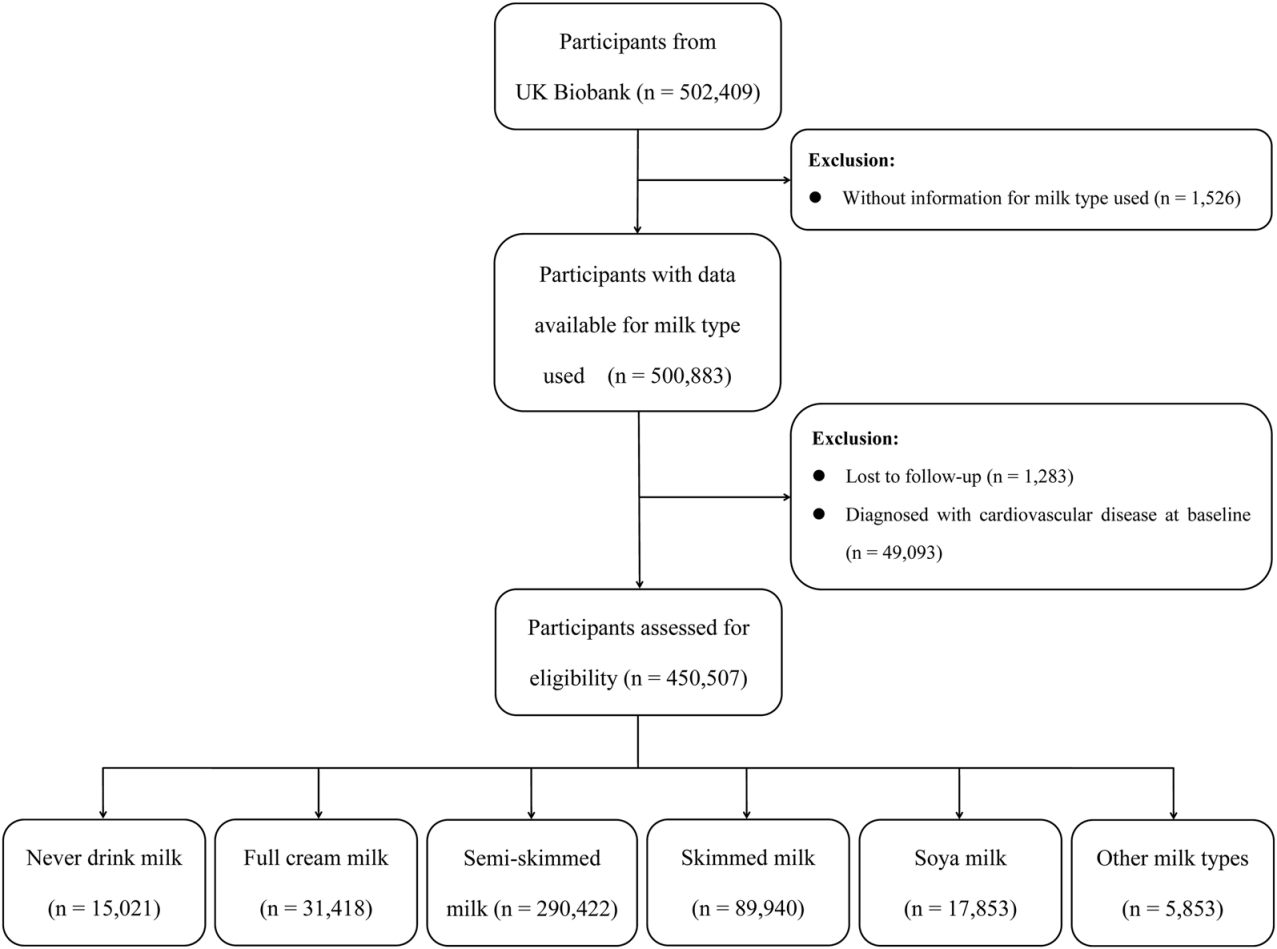
Flowchart of participant selection

The UK Biobank included 502,409 participants from England, Scotland and Wales between March 2006 and July 2010. Inclusion criteria included age 40–69 years and living within a reasonable travel distance (10 miles) from one of the 22 assessment centers. Participants provided biological samples, physical measurements and baseline information in the assessment centers. After 6 participants dropped out of the UK Biobank project, 502,409 were obtained. Furthermore, 1526 participants without information for milk type used, 1283 participants lost to follow-up and 49,093 participants diagnosed with CVD at baseline were excluded. Then, 450,507 participants without CVD at baseline were enrolled in this study (Fig. 1).

### Exposure assessment

All the enrolled participants attended one of 22 assessment centers across the UK and finished a questionnaire using a touch screen device. One of the questions asked, “What type of milk do you mainly use?” The participants can choose their answer from a list of milk, including skimmed, semi-skimmed, full cream, soy and other milk types.

### Assessment of outcomes

The study’s main outcomes were all-cause mortality, CVD mortality and CVD events. The secondary outcomes were stroke and myocardial infarction incidence and mortality. The date and cause of death were determined by linking to the Death Registry of the National Health Service (NHS) Information Centre for the UK and Welsh participants and the Death Registry of the Scottish NHS Central Registry for Scottish participants. Additionally, dates and reasons for hospitalization were identified by linking to Scottish morbidity records for Scottish participants and health event statistics for England and Wales participants. Detailed information can be found at http://content.digital.nhs.uk/services. This date was used as the end date for follow-up unless death or hospital admission occurred first for CVD outcomes. The CVD events were considered as hospitalization or death due to the following ICD-10 codes according to the hospital or death record: CVD death codes: I00–I99, CVD codes: I20–I25 and I60–I64, myocardial infarction codes: I21, I22, I23, I24.1 or I25.2 and stroke codes: I60–I64 [15–18].

### Other variables

The baseline questionnaires were used to evaluate some potential confounding variables: sociodemographic factors (age, sex, ethnic background and household income), socioeconomic status (Townsend Deprivation Index), lifestyle habits (smoking status, alcohol consumption, tea intake, processed meat intake obesity, dietary intake (fruits and vegetables). Furthermore, medication use (blood pressure drugs and cholesterol-lowering drugs use), vitamin supplements (vitamin A, vitamin B, vitamin C, vitamin D, vitamin E, multivitamins, or folic acid), minerals supplements (calcium, iron, zinc or selenium) and comorbidities (hypertension, diabetes, high cholesterol and long-term illness) were evaluated. The Townsend Deprivation Index was provided in the UK Biobank. The information for medical history (hypertension, diabetes, high cholesterol and long-term illness) was obtained through self-report at baseline. The body mass index (BMI) was calculated as weight in kilograms divided by height in meters squared. In this study, obesity was considered as BMI ≥ 30, which consists with the European guidelines for obesity management in adults [19]. Details of these evaluations could be obtained from UK Biobank (www.ukbiobank.ac.uk).

### Statistical analysis

Lilliefors test was adopted to detect if the data have a normal distribution [20, 21]. Continuous variables were indicated as mean ± standard deviation for normally distributed data; otherwise, they were shown as median and interquartile range. All categorical variables were presented as counts with percentages. Cox regression models were adopted to analyze the connection between milk consumption and all-cause mortality and CVD outcomes (CVD mortality, myocardial infarction mortality, stroke mortality, CVD events, myocardial infarction and stroke). Two models were constructed, and the basic model was adjusted for baseline age (years) and sex (male or female). The multivariable model was further adjusted for Townsend Deprivation Index, ethnic background (white or others), household income (< £18,000, £18,000–£30,999, £31,000–£51,999, £52,000–£100,000, or > £100,000), obesity (yes or no), fruit consumption, vegetable consumption, smoking status (current, never or previous). In addition, alcohol intake (< 1, 1–2, 3–4, or > 4 times/week), tea intake (< 2.0, 2.0–3.9, or ≥ 4.0 servings/day), processed meat intake (< 2, 2–4, 5–6, or > 6 times/week), vitamin use (yes or no), minerals use (yes or no), blood pressure drugs use (yes or no), cholesterol-lowering drugs use (yes or no), hypertension (yes or no), diabetes (yes or no), high cholesterol (yes or no) and longstanding illness (yes or no).

Additionally, several subgroup analyses were conducted by age (≥ 60 vs < 60 years), ethnic background (white vs others), sex (male vs female), current smoking status (yes vs no), diabetes (yes vs no), high cholesterol (yes vs no), hypertension (yes vs no), obesity (yes vs no), longstanding illness (yes vs no), blood pressure drugs use (yes vs no), cholesterol-lowering drugs use (yes vs no) and minerals use (yes vs no).

### Sensitivity analysis

Several sensitivity analyses were conducted to determine the stability of cox regression model results. First, the participants who had undergone an outcome event during the first 2 years of follow-up were removed. Second, the participants who use vitamin were excluded. Third, all missing covariate data were imputed using multiple imputations. Fourth, the participants diagnosed with cancer at baseline were removed. All results were indicated as HR and 95% CI. R version 4.1.2 (www.r-project.org) was adopted for the analysis, and a two-sided *P* value < 0.05 was set as statistically significant.

## Results

### Characteristics of participants at baseline

The median age of participants was 57 years (interquartile range 50.00–63.00); 250,478 (55.6%) were women, 423,954 (94.1%) were white, and 435,486 (96.7%) were milk consumers. Full cream, semi-skimmed, skimmed, soy and other milk were used by 31,418, 290,422, 89,940, 17,853, and 5,853 participants, respectively. Semi-skimmed milk (64.5%) was the most commonly consumed, followed by skimmed (20.0%), full cream (7.0%), soy (4.0%) and others (1.3%). Skimmed milk consumers were more likely to be women, white, obese, former smokers, blood pressure drug users, cholesterol-lowering drug users, and in poor health. The baseline characteristics of enrolled participants are indicated in Table 1.

**Table 1:** Baseline features of participants

Characteristics	Overall (n = 450,507)	Never (n = 15,021)	Milk type used				
			Full cream (n = 31,418)	Semi-skimmed (n = 290,422)	Skimmed (n = 89,940)	Soya (n = 17,853)	Other (n = 5,853)
Follow-up time (median [IQR])	12.1 [11.4–12.8]	12.1 [11.4–12.8]	12.1 [11.3–12.9]	12.1 [11.4–12.8]	12.1 [11.4–12.8]	12.1 [11.4–12.8]	12.0 [11.2–12.7]
Age (median [IQR])	57.00 [50.00, 63.00]	57.00 [49.00, 62.00]	56.00 [48.00, 62.00]	57.00 [49.00, 63.00]	59.00 [51.00, 63.00]	57.00 [50.00, 62.00]	56.00 [48.00, 62.00]
Female	250,478 (55.6)	8803 (58.6)	12,585 (40.1)	154,237 (53.1)	58,365 (64.9)	12,965 (72.6)	3,523 (60.2)
Townsend Deprivation Index (median [IQR])	− 2.17 [− 3.67, 0.46]	− 1.46 [− 3.38, 1.63]	− 0.97 [− 3.10, 2.25]	− 2.24 [− 3.69, 0.31]	− 2.41 [− 3.78, − 0.10]	− 1.96 [− 3.55, 0.81]	− 1.06 [− 3.22, 2.22]
Ethnic background							
White	423,954 (94.1)	13,866 (92.3)	27,862 (88.7)	274,914 (94.7)	85,717 (95.3)	16,387 (91.8)	5208 (89.0)
Other	26,552 (5.9)	1155 (7.7)	3556 (11.3)	15,507 (5.3)	4223 (4.7)	1466 (8.2)	645 (11.0)
Household income (￡)							
< 18,000	83,165 (21.7)	2891 (22.5)	8398 (31.7)	51,388 (20.7)	15,696 (20.6)	3327 (22.0)	1465 (31.4)
18,000–30,999	96,658 (25.2)	2971 (23.1)	6983 (26.4)	62,007 (25.0)	19,547 (25.7)	3996 (26.5)	1154 (24.7)
31,000–51,999	101,625 (26.5)	3273 (25.5)	6046 (22.9)	66,964 (27.0)	20,213 (26.6)	4039 (26.8)	1090 (23.4)
52,000–100,000	80,437 (21.0)	2817 (21.9)	4009 (15.2)	53,645 (21.6)	16,221 (21.3)	2995 (19.8)	750 (16.1)
> 100,000	21,604 (5.6)	892 (6.9)	1018 (3.8)	14,349 (5.8)	4403 (5.8)	735 (4.9)	207 (4.4)
Obesity	105,752 (23.6)	3851 (25.8)	6003 (19.3)	68,178 (23.6)	23,574 (26.3)	2905 (16.4)	1241 (21.4)
Fruit consumption (median [IQR])	2.00 [1.00, 3.00]	2.00 [1.00, 3.00]	1.00 [1.00, 2.00]	2.00 [1.00, 3.00]	2.00 [1.00, 3.00]	2.00 [2.00, 3.00]	2.00 [1.00, 3.00]
Vegetable consumption (median [IQR])	2.00 [2.00, 3.00]	3.00 [2.00, 4.00]	2.00 [1.00, 3.00]	2.00 [2.00, 3.00]	3.00 [2.00, 3.00]	3.00 [2.00, 4.00]	3.00 [2.00, 4.00]
Current smoking status							
Current	47,050 (10.5)	2148 (14.4)	6550 (20.9)	30,229 (10.4)	6409 (7.2)	968 (5.4)	746 (12.8)
Never	250,282 (55.8)	7404 (49.5)	16,119 (51.6)	162,858 (56.3)	50,195 (56.0)	10,510 (59.0)	3196 (55.0)
Previous	151,541 (33.8)	5405 (36.1)	8599 (27.5)	96,323 (33.3)	33,014 (36.8)	6327 (35.5)	1873 (32.2)
Alcohol intake (times/week)							
< 1	136,735 (30.4)	4664 (31.1)	11,338 (36.1)	83,071 (28.7)	28,070 (31.3)	7021 (39.3)	2571 (44.0)
1–2	116,661 (25.9)	3108 (20.7)	6999 (22.3)	76,734 (26.4)	23,949 (26.6)	4546 (25.5)	1325 (22.7)
3–4	104,899 (23.3)	3209 (21.4)	5848 (18.7)	70,036 (24.1)	21,022 (23.4)	3676 (20.6)	1108 (19.0)
> 4	91,817 (20.4)	4026 (26.8)	7167 (22.9)	60,355 (20.8)	16,836 (18.7)	2597 (14.6)	836 (14.3)
Tea intake (median [IQR])	3.00 [1.00, 5.00]	1.00 [0.00, 3.00]	3.00 [1.00, 5.00]	3.00 [2.00, 5.00]	3.00 [2.00, 5.00]	3.00 [1.00, 5.00]	3.00 [1.00, 5.00]
Processed meat intake (times/week)							
< 2	311,052 (69.2)	10,688 (71.5)	18,435 (58.9)	195,549 (67.4)	67,033 (74.6)	15,111 (84.8)	4236 (72.7)
2–4	120,878 (26.9)	3490 (23.3)	10,527 (33.7)	82,877 (28.6)	20,207 (22.5)	2420 (13.6)	1357 (23.3)
5–6	13,945 (3.1)	560 (3.7)	1722 (5.5)	9210 (3.2)	2049 (2.3)	232 (1.3)	172 (2.9)
> 6	3640 (0.8)	230 (1.5)	579 (1.9)	2207 (0.8)	504 (0.6)	54 (0.3)	66 (1.1)
Supplement or drug use							
Vitamin use	142,913 (32.1)	5339 (35.9)	8288 (26.8)	86,463 (30.1)	30,908 (34.8)	9225 (52.1)	2690 (46.7)
Minerals use	192,167 (43.1)	6221 (41.8)	10,356 (33.4)	11,9542 (41.6)	42,568 (47.8)	10,542 (59.5)	2938 (50.9)
Blood pressure drugs use	81,767 (18.4)	2749 (18.5)	4359 (14.2)	52,700 (18.4)	18,459 (20.8)	2560 (14.5)	940 (16.4)
Cholesterol lowering drugs use	63,807 (14.4)	2048 (13.8)	2810 (9.1)	41,092 (14.4)	15,260 (17.2)	1948 (11.0)	649 (11.3)
Health conditions							
Hypertension	113,141 (25.1)	3951 (26.3)	6528 (20.8)	72,757 (25.1)	24,801 (27.6)	3,721 (20.8)	1,383 (23.6)
Diabetes	21,398 (4.7)	690 (4.6)	1231 (3.9)	14,062 (4.8)	4531 (5.0)	626 (3.5)	258 (4.4)
High cholesterol	49,281 (10.9)	1594 (10.6)	2053 (6.5)	31,334 (10.8)	12,073 (13.4)	1706 (9.6)	521 (8.9)
Longstanding illness	82,347 (18.6)	3181 (21.6)	5850 (19.0)	50,821 (17.8)	17,367 (19.7)	3735 (21.3)	1393 (24.6)

Values were numbers (percentages) unless stated otherwise

### Association between milk consumption and all-cause mortality

The median follow-up time was 12.1 years (IQR, 11.4 to 12.8 years). During the follow-up period, 21,119 deaths were observed, including 3,679 CVD, 810 myocardial infarction, and 718 stroke deaths. The basic model with adjusted age and sex found that semi-skimmed, skimmed, and soy milk consumption was connected to a lower risk of all-cause mortality, CVD mortality, myocardial infarction mortality and CVD events.

After adjusting for age, sex, and Townsend Deprivation Index, etc., the adjusted hazard ratios of association between milk consumption and all-cause mortality were 0.84 (95% CI 0.79 to 0.91; P = 0.000) for semi-skimmed milk; 0.82 (0.76 to 0.88; P = 0.000) for skimmed milk and 0.83 (0.75 to 0.93; P = 0.001) for soy milk. However, no significant correlation was observed between milk consumption and death for full cream milk (HR 1.05; 95% CI 0.97 to 1.14; P = 0.209) and other milk (HR 1.09; 95% CI 0.96 to 1.24; P = 0.163).

### Milk consumption and CVD outcomes

A total of 810 myocardial infarction deaths, 6443 myocardial infarction events, 718 stroke deaths and 6291 stroke events were recorded. As shown in Table 2, that semi-skimmed milk consumption was significantly associated with lower risks of CVD mortality (HR 0.75, 95% CI 0.63 to 0.88; P = 0.000), CVD events (HR 0.89, 95% CI 0.81 to 0.97; P = 0.007), stroke mortality (HR 0.70, 95% CI 0.49 to 1.00; P = 0.048) and stroke (HR 0.82, 95% CI 0.72 to 0.93; P = 0.002). Similarly, skimmed milk was found related to lower risks of CVD mortality (HR 0.74, 95% CI 0.62 to 0.88; P = 0.001), CVD events (HR 0.87, 95% CI 0.79 to 0.96; P = 0.003), and stroke (HR 0.80, 95% CI 0.70 to 0.92; P = 0.001). Soy milk was observed to be connected to lower risks of CVD mortality (HR 0.67, 95% CI 0.51 to 0.86; P = 0.002) CVD events (HR 0.82, 95% CI 0.72 to 0.93; P = 0.002), and stroke (HR 0.76, 95% CI 0.63 to 0.92; P = 0.004). However, no significant associations were found between full cream milk or other milk consumption and CVD outcomes (P > 0.05).

**Table 2:** Associations of milk type used with risk of all cause mortality and cardiovascular outcomes. Values were numbers (percentages) unless stated otherwise

Outcome	Never	Milk type used, hazard ratios (95% confidence interval)				
		Full cream	Semi-skimmed	Skimmed	Soya	Other
All cause mortality						
Event, n (%)	1087 (7.24)	2787 (8.87)	17,524 (6.03)	5119 (5.69)	859 (4.81)	478 (8.17)
Basic model^a^	1 (Reference)	1.15 (1.07–1.24)	0.79 (0.74–0.84)	0.74 (0.69–0.79)	0.71 (0.65–0.77)	1.16 (1.04–1.29)
Multivariable model^b^	1 (Reference)	1.05 (0.97–1.14)	0.84 (0.79–0.91)	0.82 (0.76–0.88)	0.83 (0.75–0.93)	1.09 (0.96–1.24)
CVD mortality						
Event, n (%)	208 (1.38)	527 (1.68)	3087 (1.06)	887 (0.99)	113 (0.63)	95 (1.62)
Basic model^a^	1 (Reference)	1.06 (0.90–1.24)	0.71 (0.61–0.81)	0.69 (0.59–0.80)	0.52 (0.41–0.65)	1.21 (0.95–1.54)
Multivariable model^b^	1 (Reference)	0.93 (0.77–1.12)	0.75 (0.63–0.88)	0.74 (0.62–0.88)	0.67 (0.51–0.86)	0.91 (0.67–1.24)
Myocardial infarction mortality						
Event, n (%)	47 (0.31)	110 (0.35)	672 (0.23)	180 (0.20)	28 (0.16)	12 (0.21)
Basic model^a^	1 (Reference)	0.91 (0.65–1.28)	0.67 (0.50–0.90)	0.64 (0.46–0.88)	0.61 (0.38–0.98)	0.68 (0.36–1.28)
Multivariable model^b^	1 (Reference)	0.87 (0.58–1.29)	0.74 (0.52–1.04)	0.70 (0.48–1.01)	0.77 (0.45–1.33)	0.45 (0.19–1.06)
Stroke mortality						
Event, n (%)	41 (0.27)	80 (0.25)	613 (0.21)	210 (0.23)	36 (0.20)	14 (0.24)
Basic model^a^	1 (Reference)	0.95 (0.65–1.38)	0.74 (0.54–1.02)	0.77 (0.55–1.08)	0.74 (0.47–1.16)	0.89 (0.49–1.64)
Multivariable model^b^	1 (Reference)	0.76 (0.50–1.17)	0.70 (0.49–1.00)	0.76 (0.52–1.11)	0.80 (0.49–1.32)	0.48 (0.20–1.14)
CVD events						
Event, n (%)	712 (5.49)	1756 (6.55)	12,587 (5.03)	3519 (4.48)	533 (3.45)	279 (5.43)
Basic model^a^	1 (Reference)	1.05 (0.96–1.14)	0.85 (0.79–0.92)	0.80 (0.74–0.87)	0.70 (0.62–0.78)	1.02 (0.89–1.17)
Multivariable model^b^	1 (Reference)	0.97 (0.88–1.07)	0.89 (0.81–0.97)	0.87 (0.79–0.96)	0.82 (0.72–0.93)	0.93 (0.79–1.10)
Myocardial infarction						
Event, n (%)	245 (1.89)	664 (2.48)	5390 (2.15)	1478 (1.88)	235 (1.52)	90 (1.75)
Basic model^a^	1 (Reference)	1.09 (0.94–1.26)	1.07 (0.94–1.21)	1.03 (0.90–1.18)	0.95 (0.79–1.13)	0.94 (0.74–1.20)
Multivariable model^b^	1 (Reference)	1.01 (0.85–1.19)	1.07 (0.93–1.24)	1.08 (0.93–1.26)	1.04 (0.85–1.28)	0.85 (0.64–1.14)
Stroke						
Event, n (%)	333 (2.57)	713 (2.67)	5208 (2.08)	1516 (1.93)	244 (1.58)	126 (2.46)
Basic model^a^	1 (Reference)	0.95 (0.83–1.08)	0.77 (0.69–0.86)	0.72 (0.64–0.82)	0.66 (0.56–0.78)	0.97 (0.79–1.18)
Multivariable model^b^	1 (Reference)	0.91 (0.78–1.06)	0.82 (0.72–0.93)	0.80 (0.70–0.92)	0.76 (0.63–0.92)	0.99 (0.78–1.25)

*CVD* cardiovascular disease

^a^Basic model: adjusted for baseline age (years) and sex (male or female)

^b^Multivariable model: adjusted for baseline age (years) and sex (male or female), Townsend Deprivation Index, ethnic background (white or others), household income (< £18,000, £18,000–£30,999, £31,000–£51,999,£52,000–£100,000, or > £100,000), obesity (yes or no), fruit consumption (< 2.0, 2.0–3.9, or ≥ 4.0 servings/day), vegetable consumption (< 2.0, 2.0–3.9, or ≥ 4.0 servings/day), smoking status (current, never or previous), alcohol intake (< 1, 1–2, 3–4, or > 4 times/ week), tea intake (< 2.0, 2.0–3.9, or ≥ 4.0 servings/day), processed meat intake (< 2, 2–4, 5–6, or > 6 times/week), vitamin use (yes or no), minerals use (yes or no), blood pressure drugs use (yes or no), cholesterol lowering drugs use (yes or no), hypertension (yes or no), diabetes (yes or no), high cholesterol (yes or no) and longstanding illness (yes or no)

### Subgroup analysis

**Fig. 2:**
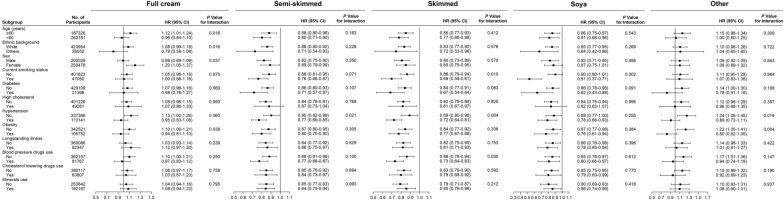
Association of milk consumption and the risk of all cause mortality stratified by potential risk factors. The results were adjusted for baseline age (years) and sex (male or female), Townsend Deprivation Index, ethnic background (white or others), household income (< £18,000, £18,000–£30,999, £31,000–£51,999, £52,000–£100,000, or > £100,000), obesity (yes or no), fruit consumption, vegetable consumption, smoking status (current, never or previous), alcohol intake (< 1, 1–2, 3–4, or > 4 times/week), tea intake (< 2.0, 2.0–3.9, or ≥ 4.0 servings/day), processed meat intake (< 2, 2–4, 5–6, or > 6 times/week), vitamin use (yes or no), minerals use (yes or no), blood pressure drugs use (yes or no), cholesterol lowering drugs use (yes or no), hypertension (yes or no), diabetes (yes or no), high cholesterol (yes or no) and longstanding illness (yes or no)

Stratified analysis was performed to observe the correlation between milk consumption and risk of all-cause mortality and CVD outcomes via multivariable model. Full cream milk consumption was connected to an increased risk of all-cause mortality (HR 1.20, 95% CI 1.05 to 1.37; P = 0.007) in participants who were women or with no hypertension (HR 1.13, 95% CI 1.02 to 1.26; P = 0.019). Moreover, other milk consumption was related to a higher risk of all-cause mortality in participants with no hypertension (HR 1.24, 95% CI 1.06 to 1.45; P = 0.008), not obese (HR 1.22, 95% CI 1.05 to 1.41; P = 0.008), not using blood pressure drugs (HR 1.17, 95% CI 1.01 to 1.36; P = 0.043) (Fig. 2).

The association between semi-skimmed milk intake and all cause mortality was stronger among participants with hypertension (P for interaction = 0.021), and connection of skimmed milk consumption with all-cause mortality was stronger among smokers (P for interaction = 0.010), hypertensive participants (P for interaction = 0.004) and blood pressure drug users (P for interaction = 0.030) for all-cause mortality. Additionally, the relationship between soy milk consumption and all-cause mortality was stronger among smokers (P for interaction = 0.002).

**Fig. 3:**
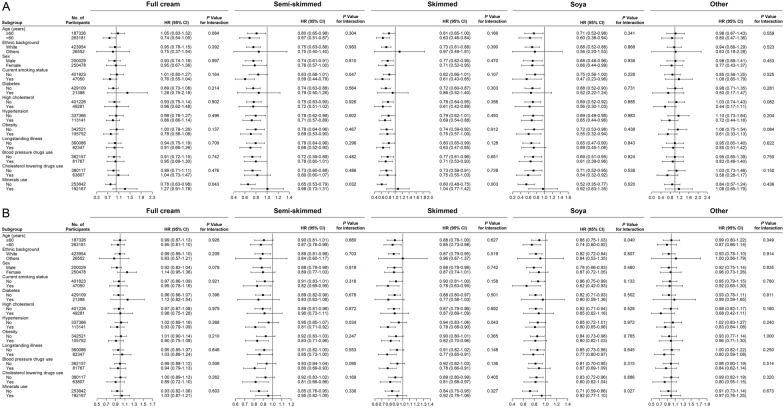
Associations of milk consumption and the risk of (A) cardiovascular mortality and (B) cardiovascular events stratified by potential risk factors. The results were adjusted for baseline age (years) and sex (male or female), Townsend Deprivation Index, ethnic background (white or others), household income (< £18,000, £18,000–£30,999, £31,000–£51,999, £52,000–£100,000, or > £100,000), obesity (yes or no), fruit consumption, vegetable consumption, smoking status (current, never or previous), alcohol intake (< 1, 1–2, 3–4, or > 4 times/week), tea intake (< 2.0, 2.0–3.9, or ≥ 4.0 servings/ day), processed meat intake (< 2, 2–4, 5–6, or > 6 times/ week), vitamin use (yes or no), minerals use (yes or no), blood pressure drugs use (yes or no), cholesterol lowering drugs use (yes or no), hypertension (yes or no), diabetes (yes or no), high cholesterol (yes or no) and longstanding illness (yes or no)

The association between semi-skimmed milk consumption and CVD outcomes was stronger among smokers (P for interaction = 0.047) and non-minerals users (P for interaction = 0.032) for CVD mortality and hypertensive participants (P for interaction = 0.034) for CVD events. Skimmed milk consumption and CVD outcomes were stronger among participants not on mineral supplements (P for interaction = 0.003) for CVD mortality and hypertension (P for interaction = 0.043) for CVD events. The connection between soy milk consumption and CVD outcomes was stronger among participants not using mineral supplements (P for interaction = 0.020) for CVD mortality and younger than 60 years old (P for interaction = 0.040 and not using mineral supplements (P for interaction = 0.027) for CVD events. We did not observe other significant interactions (all P for interaction ≥ 0.05). The associations of milk consumption with all-cause mortality and CVD outcomes were not modified by any other risk factors (Figs. 2 and 3).

### Sensitivity analysis

The relationship of milk consumption with decreased risk of all-cause mortality did not change significantly after removing participants who developed CVD events or died during the first two years of follow-up (Additional file 1: Table S1). When the participants who took vitamin were excluded, the results did not change appreciably, but full cream, semi-skimmed, skimmed, and soy milk were related to lower risks of stroke mortality (Additional file 1: Table S2). Then, the result remained stable when all the missing covariate data were imputed using multiple imputations (Additional file 1: Table S3). Finally, when the participants diagnosed with cancer at baseline were removed, the result remained stable (Additional file 1: Table S4).

## Discussion

Milk is an important food source that positively affects nutrient and energy intake, but heavy metals can counteract these benefits and affect human health. For example, the toxic trace elements lead (Pb) and cadmium (Cd) [22] can disturb the trace mineral composition of milk and negatively affect its nutritional quality [23]. Previous reports showed the presence of heavy metals in milk collected from different regions of the world [24, 25]. According to previous studies, the concentrations of Pb and Cd in milk were generally higher in developing countries but lower in developed countries, heavy metals such as Pb and Cd. Both of Pb and Cd can cross the placental barrier, which may affect the fetal brain differentiation, leading to neurotoxic effects, including memory loss and language impairment [26, 27].

Milk is rich in protein, amino acids, minerals and trace elements. Milk is a good nutrient for human health [11]. In this prospective cohort study with 450,507 participants recruited from UK Biobank between 2006 and 2010, it was observed that compared with non-milk users, semi-skimmed, skimmed and soya milk consumption was related to a 16%, 18% and 17% lower risk of all-cause mortality, respectively. Additionally, semi-skimmed skimmed and soya milk consumption was significantly correlated with decreased CVD mortality, CVD events and stroke risks. The protective association of skimmed milk consumption against all-cause mortality was stronger in smokers and blood pressure medication users. Association of semi-skimmed, skimmed and soya milk consumption with CVD mortality was stronger among participants not on mineral supplements. Correlation of semi-skimmed and skimmed milk with CVD events was stronger among participants with hypertension.

The association of milk consumption with all-cause mortality and CVD outcomes has been controversial [7, 13, 28]. Xu et al. reported that participants with low fat milk intake indicated a decreased risk of mortality compared to participants consuming whole milk [11]. Similarly, Wang et al. found that skimmed milk was related to lower risk of mortality compared to whole milk [8], while another study using two large Swedish cohorts showed that high milk consumption was connected to higher mortality [12]. In the present study, we observed that full cream milk was not related to all-cause mortality and CVD outcomes, while semi-skimmed and skimmed milk were connected to lower all-cause mortality risk, CVD mortality and incidence of CVD. Skimmed milk showed better protection than semi-skimmed milk. Studies have shown that increased intake of low-fat dairy was associated with lower CVD mortality, but not high-fat dairy intake [29]. Consumption of low-fat dairy products was related to a reduction in hypertension risk and serum LDL-C [29], which may account for the potential CVD benefits of low-fat milk. In this study, low-fat milk consumers were more likely to be obese (41.5%), possibly because obese people tended to choose skim milk. A previous randomized controlled trial showed no significant effect of low-fat milk or whole cheese consumption on ambulatory blood pressure compared with a dairy-free diet [30]. However, compared with a low-fat milk diet, consumption of regular-fat cheese increased LDL particle size, which may be associated with CVD events [31].

The connection between skimmed milk consumption and CVD outcomes in this study was stronger among participants with hypertension for CVD events. A previous study indicated that hypertension is a risk factor for CVD [32]. It was also related to hyperlipidemia, with many common risk factors [33]. Skimmed milk may provide more benefits for hypertensive patients.

Previous studies have shown that dietary habits were one of the major determinants of CVD, responsible for approximately half of deaths from cardiometabolic disease [34]. Skimmed milk may show better results against CVD outcomes compared to full cream milk. At the same time, milk with different fat levels exhibits different acidity and stability, casein content, total solids content and complex interactions [8]; thus, skimmed milk benefits may not be solely attributable to lower milk fat intake [35].

A previous study indicated that skimmed milk presented higher inhibitory activities for oxidative damage of deoxyribose [36]. Additionally, another report showed that soya milk consumption could lead to decreased oxidative damage for DNA bases [37]. Moreover, skimmed milk proteins exhibited ability of good free-radical quenching [38]. Antioxidant effect of milk may be protective against cardiovascular disease, while the protective association of semi-skimmed, skimmed, and soy milk with lower risks of all-cause mortality and CVD outcomes needs to be further explored.

In this study, soy milk consumption was related to 17%, 33%, 18% and 24% lower risk of all-cause mortality, CVD mortality, CVD events and stroke, respectively. Soy milk showed a better reduction in CVD outcomes than semi-skimmed and skimmed milk. Previous clinical trials indicated that soy milk consumption positively affected several cardiovascular events [39, 40]. Positive health effects of whole soy products, such as soy milk, have also been documented [41, 42]; a previous study indicated that soy milk consumption could decrease triglycerides and systolic blood pressure levels. Furthermore, soy peptides could act as inhibitory peptides for angiotensin-converting enzymes, leading to a hypotensive effect [43]. The renin-angiotensin system plays a crucial role in the regulation of renal, cardiac and vascular physiology. Activation of the renin-angiotensin system is a major cause of many common pathological conditions, including hypertension, heart failure, and renal disease. Metabolites of angiotensin I and II were initially considered biologically inactive, but there is now increasing evidence that they play key roles in cardiovascular physiology and pathophysiology [44].

## Strengths and limitations

This study had several strengths, including the large sample size, minimal loss to follow-up and population-based cohort study design. However, there are several limitations. First, we adjusted the potential confounders; however, residual confounding can not be completely ruled out. Second, this study is observational, and no causality was obtained. Third, this study did not record detailed information on the dose and duration of milk use, so dose–response associations between milk consumption cannot be assessed. Fourth, due to the low response rate, potential selection biases may limit the generalizability of the conclusions in the wider UK population.

## Conclusions

A total of 450,507 participants without CVD at baseline were enrolled in this study, and 435,486 (96.7%) were milk consumers. Compared with non-milk users, semi-skimmed, skimmed, and soy milk consumption was related to a lower risk of all-cause mortality and CVD outcomes. Skim milk consumption was more beneficial for all-cause mortality, while soy milk consumption was more beneficial for CVD events. This study recommends skimmed or soy milk over full cream milk to improve clinical outcomes. Additionally, the difference between skimmed and full cream milk should be discovered for the actual reason for this impact. For this purpose, ingredient analyses such as mass spectrum need to be conducted, and well-designed biological experiments should be performed based on the results.

## Supplementary Information

**Additional file 1: Table S1.** Associations of milk consumption with the risk of all-cause mortality and CVD outcomes after excluding participants who experienced an outcome event during the first two years of follow-up (n = 448,657). **Table S2.** Associations of milk consumption with the risk of all-cause mortality and CVD outcomes after excluding participants who use vitamin (n = 307,594). **Table S3.** Associations of milk consumption with the risk of all-cause mortality and CVD outcomes with all missing covariate data imputed using multiple imputation (n = 450,507). **Table S4.** Associations of milk consumption with the risk of all-cause mortality and CVD outcomes after excluding participants who were diagnosed with cancer at baseline (n = 413,422).

## Data Availability

The datasets used and/or analyzed during the current study are available from the corresponding author upon reasonable request.
