# Supplementary material for: Association of milk consumption with all-cause mortality and cardiovascular outcomes: a UK Biobank based large population cohort study
Source: J Transl Med. 2023 Feb 18;21:130. doi: 10.1186/s12967-023-03980-4 (PMC9938581; doi:10.1186/s12967-023-03980-4)
Supplement: Supplementary file 1 — Additional file 1: Table S1. Associations of milk consumption with the risk of all-cause mortality and CVD outcomes after excluding participants who experienced an outcome event during the first two years of follow-up (n = 448,657). Table S2. Associations of milk consumption with the risk of all-cause mortality and CVD outcomes after excluding participants who use vitamin (n = 307,594). Table S3. Associations of milk consumption with the risk of all-cause mortality and CVD outcomes with all missing covariate data imputed using multiple imputation (n = 450,507). Table S4. Associations of milk consumption with the risk of all-cause mortality and CVD outcomes after excluding participants who were diagnosed with cancer at baseline (n = 413,422). [file 12967_2023_3980_MOESM1_ESM.docx]

Table S1. Associations of milk consumption with the risk of all-cause mortality and CVD outcomes after excluding participants who experienced an outcome event during the first two years of follow-up (n=448,657).

| **Outcome** | **Never** | **Milk type used, hazard ratios (95% confidence interval)^#^** | | | | |
| --- | --- | --- | --- | --- | --- | --- |
|  |  | **Full cream** | **Semi-skimmed** | **Skimmed** | **Soya** | **Other** |
| All cause mortality | 1 (Reference) | 1.04 (0.96-1.13) | 0.84 (0.78-0.91) | 0.81 (0.75-0.88) | 0.83 (0.74-0.92) | 1.09 (0.96-1.25) |
| Cardiovascular mortality | 1 (Reference) | 0.92 (0.76-1.12) | 0.76 (0.64-0.90) | 0.77 (0.64-0.92) | 0.69 (0.52-0.90) | 0.98 (0.71-1.34) |
| Myocardial infarction mortality | 1 (Reference) | 0.96 (0.62-1.48) | 0.80 (0.55-1.17) | 0.80 (0.53-1.20) | 0.79 (0.44-1.44) | 0.55 (0.23-1.32) |
| Stroke mortality | 1 (Reference) | 0.69 (0.44-1.07) | 0.66 (0.46-0.94) | 0.73 (0.50-1.08) | 0.82 (0.50-1.36) | 0.49 (0.21-1.17) |
| Cardiovascular events | 1 (Reference) | 0.97 (0.88-1.07) | 0.90 (0.82-0.98) | 0.88 (0.80-0.97) | 0.83 (0.73-0.94) | 0.95 (0.81-1.13) |
| Myocardial infarction | 1 (Reference) | 1.01 (0.86-1.20) | 1.08 (0.93-1.24) | 1.09 (0.93-1.27) | 1.05 (0.85-1.28) | 0.86 (0.65-1.15) |
| Stroke | 1 (Reference) | 0.91 (0.78-1.06) | 0.82 (0.72-0.93) | 0.80 (0.70-0.92) | 0.75 (0.62-0.91) | 0.99 (0.78-1.26) |

^#^ Multivariable Cox regression model: adjusted for baseline age (years) and sex (male or female), Townsend Deprivation Index, ethnic background (white or others), household income (<£18,000, £18,000-£30,999, £31,000-£51,999, £52,000-£100,000, or >£100,000), obesity (yes or no), fruit consumption, vegetable consumption, current smoking status (yes or no), alcohol intake (Daily or almost daily, Never, Once or twice a week, One to three times a month, Special occasions only, Three or four times a week), tea intake (cups/ day), processed meat intake (2-4 times a week, 5-6 times a week, Less than once a week, Never, Once a week, Once or more daily), vitamin use (yes or no), minerals use (yes or no), blood pressure drugs use (yes or no), cholesterol lowering drugs use (yes or no), hypertension (yes or no), diabetes (yes or no), high cholesterol (yes or no) and longstanding illness (yes or no).

Table S2. Associations of milk consumption with the risk of all-cause mortality and CVD outcomes after excluding participants who use vitamin (n=307,594).

| **Outcome** | **Never** | **Milk type used, hazard ratios (95% confidence interval)^#^** | | | | |
| --- | --- | --- | --- | --- | --- | --- |
|  |  | **Full cream** | **Semi-skimmed** | **Skimmed** | **Soya** | **Other** |
| All cause mortality | 1 (Reference) | 1.01 (0.92-1.12) | 0.84 (0.77-0.92) | 0.80 (0.73-0.88) | 0.82 (0.71-0.94) | 1.04 (0.88-1.23) |
| Cardiovascular mortality | 1 (Reference) | 0.82 (0.65-1.02) | 0.69 (0.57-0.83) | 0.66 (0.53-0.81) | 0.59 (0.42-0.84) | 0.85 (0.58-1.25) |
| Myocardial infarction mortality | 1 (Reference) | 0.77 (0.49-1.22) | 0.67 (0.45-1.00) | 0.67 (0.43-1.01) | 0.64 (0.31-1.32) | 0.53 (0.20-1.38) |
| Stroke mortality | 1 (Reference) | 0.75 (0.44-1.25) | 0.67 (0.45-1.00) | 0.66 (0.42-1.05) | 0.63 (0.31-1.28) | 0.53 (0.18-1.53) |
| Cardiovascular events | 1 (Reference) | 0.92 (0.82-1.04) | 0.85 (0.77-0.95) | 0.85 (0.76-0.95) | 0.80 (0.68-0.95) | 0.87 (0.70-1.07) |
| Myocardial infarction | 1 (Reference) | 0.97 (0.80-1.18) | 1.02 (0.86-1.22) | 1.08 (0.90-1.30) | 1.10 (0.85-1.42) | 0.76 (0.53-1.11) |
| Stroke | 1 (Reference) | 0.90 (0.75-1.08) | 0.82 (0.70-0.96) | 0.80 (0.67-0.95) | 0.75 (0.58-0.97) | 0.93 (0.67-1.28) |

^#^ Multivariable Cox regression model: adjusted for baseline age (years) and sex (male or female), Townsend Deprivation Index, ethnic background (white or others), household income (<£18,000, £18,000-£30,999, £31,000-£51,999, £52,000-£100,000, or >£100,000), obesity (yes or no), fruit consumption, vegetable consumption, smoking status (current, never or previous), alcohol intake (<1, 1-2, 3-4, or >4 times/ week), tea intake (<2.0, 2.0-3.9, or ≥4.0 servings/ day), processed meat intake (<2, 2-4, 5-6, or >6 times/ week), vitamin use (yes or no), blood pressure drugs use (yes or no), cholesterol lowering drugs use (yes or no), hypertension (yes or no), diabetes (yes or no), high cholesterol (yes or no) and longstanding illness (yes or no).

Table S3. Associations of milk consumption with the risk of all-cause mortality and CVD outcomes with all missing covariate data imputed using multiple imputation (n=450,507).

| **Outcome** | **Never** | **Milk type used, hazard ratios (95% confidence interval)^#^** | | | | |
| --- | --- | --- | --- | --- | --- | --- |
|  |  | **Full cream** | **Semi-skimmed** | **Skimmed** | **Soya** | **Other** |
| All cause mortality | 1 (Reference) | 1.06 (0.99-1.14) | 0.84 (0.79-0.90) | 0.81 (0.76-0.87) | 0.81 (0.74-0.89) | 1.09 (0.98-1.21) |
| Cardiovascular mortality | 1 (Reference) | 0.99 (0.84-1.16) | 0.78 (0.67-0.89) | 0.78 (0.67-0.90) | 0.65 (0.51-0.82) | 1.11 (0.87-1.41) |
| Myocardial infarction mortality | 1 (Reference) | 0.85 (0.60-1.20) | 0.74 (0.53-1.00) | 0.74 (0.54-1.01) | 0.78 (0.49-1.25) | 0.61 (0.33-1.16) |
| Stroke mortality | 1 (Reference) | 0.86 (0.59-1.26) | 0.78 (0.58-0.99) | 0.86 (0.61-1.20) | 0.82 (0.52-1.29) | 0.82 (0.45-1.51) |
| Cardiovascular events | 1 (Reference) | 0.99 (0.91-1.08) | 0.89 (0.82-0.96) | 0.85 (0.78-0.92) | 0.78 (0.70-0.88) | 0.96 (0.84-1.11) |
| Myocardial infarction | 1 (Reference) | 1.00 (0.86-1.16) | 1.07 (0.94-1.22) | 1.06 (0.93-1.22) | 1.04 (0.87-1.25) | 0.88 (0.69-1.13) |
| Stroke | 1 (Reference) | 0.93 (0.82-1.07) | 0.81 (0.72-0.90) | 0.76 (0.68-0.86) | 0.71 (0.60-0.84) | 0.94 (0.76-1.15) |

^#^ Multivariable Cox regression model: adjusted for baseline age (years) and sex (male or female), Townsend Deprivation Index, ethnic background (white or others), household income (<£18,000, £18,000-£30,999, £31,000-£51,999,£52,000-£100,000, or >£100,000), obesity (yes or no), fruit consumption, vegetable consumption, smoking status (current, never or previous), alcohol intake (<1, 1-2, 3-4, or >4 times/ week), tea intake (<2.0, 2.0-3.9, or ≥4.0 servings/ day), processed meat intake (<2, 2-4, 5-6, or >6 times/ week), vitamin use (yes or no), minerals use (yes or no), blood pressure drugs use (yes or no), cholesterol lowering drugs use (yes or no), hypertension (yes or no), diabetes (yes or no), high cholesterol (yes or no) and longstanding illness (yes or no).

Table S4. Associations of milk consumption with the risk of all-cause mortality and CVD outcomes after excluding participants who were diagnosed with cancer at baseline (n=413,422).

| **Outcome** | **Never** | **Milk type used, hazard ratios (95% confidence interval)^#^** | | | | |
| --- | --- | --- | --- | --- | --- | --- |
|  |  | **Full cream** | **Semi-skimmed** | **Skimmed** | **Soya** | **Other** |
| All cause mortality | 1 (Reference) | 1.02 (0.94-1.12) | 0.84 (0.78-0.91) | 0.82 (0.76-0.89) | 0.76 (0.68-0.86) | 1.00 (0.86-1.15) |
| Cardiovascular mortality | 1 (Reference) | 0.95 (0.78-1.16) | 0.76 (0.64-0.91) | 0.76 (0.64-0.92) | 0.71 (0.54-0.93) | 0.91 (0.66-1.27) |
| Myocardial infarction mortality | 1 (Reference) | 0.90 (0.59-1.36) | 0.74 (0.52-1.07) | 0.71 (0.48-1.06) | 0.79 (0.45-1.41) | 0.41 (0.16-1.06) |
| Stroke mortality | 1 (Reference) | 0.69 (0.44-1.08) | 0.67 (0.46-0.96) | 0.71 (0.48-1.05) | 0.78 (0.46-1.31) | 0.42 (0.16-1.09) |
| Cardiovascular events | 1 (Reference) | 0.97 (0.87-1.08) | 0.89 (0.82-0.98) | 0.88 (0.80-0.97) | 0.86 (0.75-0.98) | 0.93 (0.78-1.11) |
| Myocardial infarction | 1 (Reference) | 1.02 (0.86-1.22) | 1.07 (0.92-1.25) | 1.08 (0.92-1.27) | 1.09 (0.88-1.35) | 0.92 (0.69-1.24) |
| Stroke | 1 (Reference) | 0.89 (0.76-1.05) | 0.82 (0.71-0.93) | 0.81 (0.70-0.93) | 0.79 (0.64-0.96) | 0.94 (0.72-1.22) |

^#^ Multivariable Cox regression model: adjusted for baseline age (years) and sex (male or female), Townsend Deprivation Index, ethnic background (white or others), household income (<£18,000, £18,000-£30,999, £31,000-£51,999, £52,000-£100,000, or >£100,000), obesity (yes or no), fruit consumption, vegetable consumption, smoking status (current, never or previous), alcohol intake (<1, 1-2, 3-4, or >4 times/ week), tea intake (<2.0, 2.0-3.9, or ≥4.0 servings/ day), processed meat intake (<2, 2-4, 5-6, or >6 times/ week), vitamin use (yes or no), minerals use (yes or no), blood pressure drugs use (yes or no), hypertension (yes or no), diabetes (yes or no), high cholesterol (yes or no) and longstanding illness (yes or no).
